# The *Bordetella* type III secretion system effector BteA contains a conserved N-terminal motif that guides bacterial virulence factors to lipid rafts

**DOI:** 10.1111/j.1462-5822.2009.01361.x

**Published:** 2009-08-18

**Authors:** Christopher T French, Ekaterina M Panina, Sylvia H Yeh, Natasha Griffith, Diego G Arambula, Jeff F Miller

**Affiliations:** 1 Department of Microbiology, Immunology and Molecular Genetics, David Geffen School of MedicineLos Angeles, CA 90095, USA; 2 the Molecular Biology Institute, University of CaliforniaLos Angeles, CA 90095, USA; 3 the California Nanosystems Institute, University of CaliforniaLos Angeles, CA 90095, USA

## Abstract

The *Bordetella* type III secretion system (T3SS) effector protein BteA is necessary and sufficient for rapid cytotoxicity in a wide range of mammalian cells. We show that BteA is highly conserved and functionally interchangeable between *Bordetella bronchiseptica, Bordetella pertussis* and *Bordetella parapertussis*. The identification of BteA sequences required for cytotoxicity allowed the construction of non-cytotoxic mutants for localization studies. BteA derivatives were targeted to lipid rafts and showed clear colocalization with cortical actin, ezrin and the lipid raft marker GM1. We hypothesized that BteA associates with the cytoplasmic face of lipid rafts to locally modulate host cell responses to *Bordetella* attachment. *B. bronchiseptica* adhered to host cells almost exclusively to GM1-enriched lipid raft microdomains and BteA colocalized to these same sites following T3SS-mediated translocation. Disruption of lipid rafts with methyl-β-cyclodextrin protected cells from T3SS-induced cytotoxicity. Localization to lipid rafts was mediated by a 130-amino-acid lipid raft targeting domain at the N-terminus of BteA, and homologous domains were identified in virulence factors from other bacterial species. Lipid raft targeting sequences from a T3SS effector (Plu4750) and an RTX-type toxin (Plu3217) from *Photorhabdus luminescens* directed fusion proteins to lipid rafts in a manner identical to the N-terminus of BteA.

## Introduction

Lipid membrane rafts (rafts), which are prominent targets for bacterial, viral and protozoan pathogens (Manes *et al*., 2003; Lafont *et al*., 2004; Riff *et al*., 2005; Mejia *et al*., 2008), are specialized membrane microdomains enriched in cholesterol and sphingolipids that produce ordered structures distinct from surrounding areas of disordered, predominantly unsaturated lipid species (Simons and Ikonen, 1997, Laude and Prior, 2004). The clustering of signalling proteins and lipid cofactors within rafts suggests they function as platforms where close proximity increases efficiency and specificity of signalling cascades (Hanzal-Bayer and Hancock, 2007, Viola and Gupta, 2007). Although controversy exists regarding the size, function and precise physical nature of rafts (Edidin, 2003; Munro, 2003; Laude and Prior, 2004; Skwarek, 2004), considerable evidence supports their importance for attachment of bacterial pathogens (e.g. *Mycoplasma, Shigella*, enteropathogenic *Escherichia coli*, *Porphyromonas, Yersinia*) (Lafont *et al*., 2004; Riff *et al*., 2005; Allen-Vercoe *et al*., 2006; Hawkes and Mak, 2006, Mejia *et al*., 2008; Hayward *et al*., 2009), bacterial invasion (*Shigella, Salmonella, Listeria, Mycobacterium, Chlamydia*) (Manes *et al*., 2003; Lafont *et al*., 2004; Lafont and van der Goot, 2005a), oligomerization of pore-forming toxins (aerolysin, streptolysin O, listeriolysin O) (Gekara *et al*., 2005) and cell-surface binding by A-B toxins (cholera toxin, shiga toxin) (Takenouchi *et al*., 2004; Lencer and Saslowsky, 2005). Recent evidence suggests that association of *Salmonella* and *Shigella* type III secretion system (T3SS) translocon components with target cell membranes is cholesterol-dependent (Lafont and van der Goot, 2005b). Although interactions between bacterial components and the exofacial surface of lipid rafts have been extensively studied, virulence factor targeting events occurring on the cytoplasmic side of rafts are less well understood.

In this study we report an analysis of the activity and intracellular localization of BteA, a 658 amino acid (aa) effector protein secreted by the *Bordetella bsc* T3SS (Panina *et al*., 2005; Kuwae *et al*., 2006). The *bsc* T3SS is most extensively characterized in *Bordetella bronchiseptica*, the broad-host-range evolutionary progenitor of *Bordetella pertussis* and *Bordetella parapertussis* that cause acute respiratory diseases in humans (Yuk *et al*., 1998; 2000). Although nearly identical T3SS loci are encoded in the genomes of all three species, the ability to readily detect T3SS activity *in vitro*, and the availability of natural-host animal models to study respiratory infection have made *B. bronchiseptica* the species of choice for studying type III secretion by *Bordetella*. The loci encoding *bteA* and the *bsc* T3SS apparatus are co-regulated by the alternative sigma factor BtrS, which in turn is dependent on the BvgAS phosphorelay for expression (Mattoo *et al*., 2004). In addition to transcriptional control, the partner-switching proteins BtrU, BtrV and BtrW regulate secretion through a complex series of protein–protein interactions, serine-phosphorylation and serine-dephosphorylation events (Mattoo *et al*., 2004; Kozak *et al*., 2005). BteA is an unusually potent cytotoxin capable of inducing rapid, non-apoptotic death in a diverse array of cell types, and precise, multilevel control of expression and secretion may be essential for its role in promoting persistent colonization of the respiratory epithelium (Stockbauer *et al*., 2003; Panina *et al*., 2005; Shrivastava and Miller, 2009). BteA is the only *Bordetella* effector that has been identified and its central importance is illustrated by the observation that null mutations in *bteA* recapitulate phenotypes associated with mutations that eliminate type III secretion altogether (Panina *et al*., 2005). Despite its central role in pathogenesis, little is known regarding the events that occur following BteA injection into target cells. In the course of analysing the intracellular localization of BteA, we have identified a lipid raft targeting (LRT) motif found in virulence determinants expressed by diverse bacterial pathogens.

## Results

### BteA is functionally conserved among *Bordetella* species

**Fig. 1:**
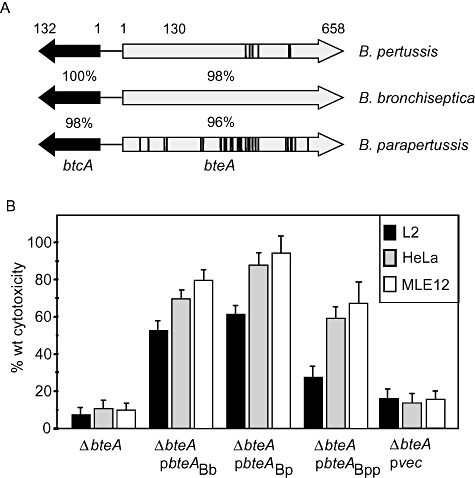
*B. pertussis*, *B. parapertussis* and *B. bronchiseptica bteA* alleles are functionally interchangeable. A. Schematic representation of *bteA* loci from three *Bordetella* species. The per cent amino acid identities between *B. bronchiseptica* proteins and their homologues in human-adapted *B. pertussis* and *B. parapertussis* are indicated. Positions of residues that differ between *B. bronchiseptica* and other species are shown by vertical bars. B. *B. pertussis* and *B. parapertussis bteA* alleles are functionally interchangeable with *B. bronchiseptica bteA*. HeLa, L2 and MLE-12 epithelial cell lines were infected with wild-type *B. bronchiseptica* RB50 (WT), a Δ*bteA* derivative (Δ*bteA*), the Δ*bteA* strain complemented with vector alone (pvec), or the Δ*bteA* strain complemented with *bteA* loci from *B. bronchiseptica* RB50 (p*bteA*_Bb_), *B. pertussis* Tohama 1 (p*bteA*_Bp_), or *B. parapertussis* 12822 (p*bteA*_Bpp_). Cytotoxicity was measured by LDH release assays performed 1 h post infection at a multiplicity of infection of 50:1.

The *bsc* T3SS loci encoded by *B. pertussis*, *B. parapertussis* and *B. bronchiseptica* are highly conserved (Yuk *et al*., 1998). *bteA* and *btcA*, which encode the BteA effector and its chaperone, respectively, are present in the genomes of all three species and are predicted to encode nearly identical proteins (Fig. 1A). As shown in Fig. 1B, *bteA* alleles from *B. pertussis* and *B. parapertussis* were able to complement a Δ*bteA* derivative of *B. bronchiseptica* strain RB50. Cytotoxicity against rat lung epithelial (L2), human epithelial (HeLa) and mouse lung epithelial (MLE12) cells was restored to levels comparable to those observed with the RB50 *bteA* allele, and the morphological characteristics of dying cells were identical (data not shown). The slightly lower levels of complementation observed with *B. parapertussis* BteA correspond to its greater degree of divergence. These results demonstrate that the BteA effector proteins encoded by *Bordetella* species are functionally conserved.

### Identification of non-cytotoxic BteA derivatives

**Fig. 2:**
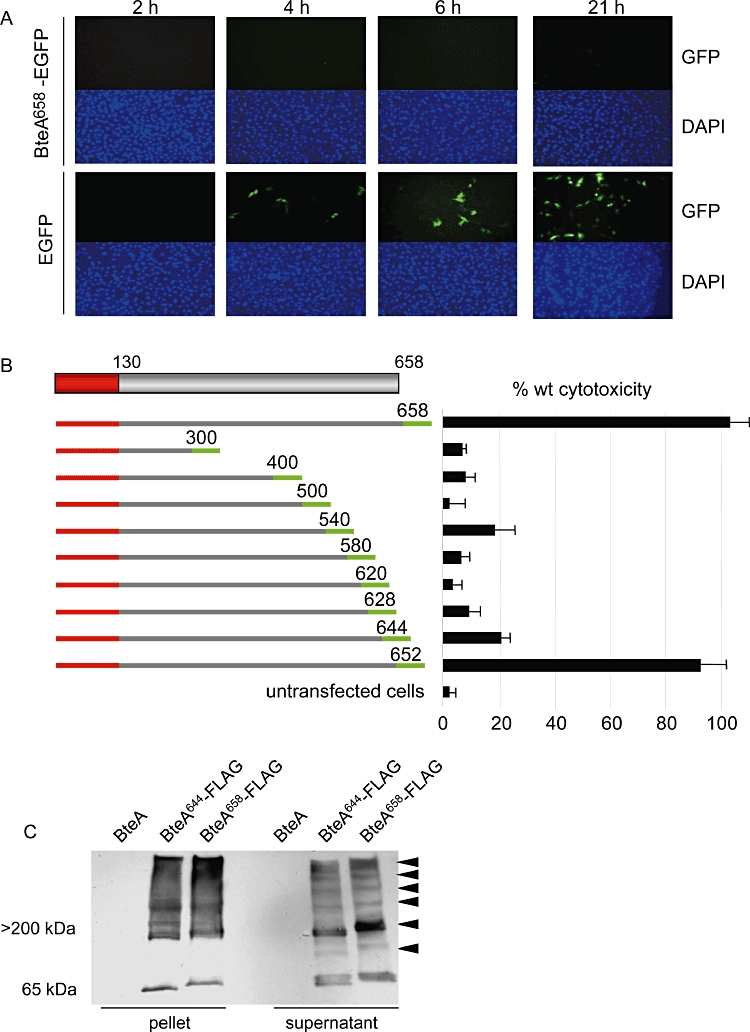
BteA is a potent cytotoxin for mammalian cells. A. Full-length BteA is not detected in eukaryotic cells by fluorescence microscopy. HeLa cells were transfected with BteA^658^-EGFP or EGFP alone for 2, 4, 6 or 21 h and visualized via fluorescence microscopy. Typical images of the same field showing green fluorescence and DAPI nuclear staining are shown. B. Full-length BteA (aa 1–658) and C-terminally truncated mutants were fused to EGFP (green bars) on the plasmid pEGFP-N1. The EGFP tag is not drawn to scale. The N-terminal BteA domain (aa 1–130) is shown in red and residues at EGFP fusion junctions are indicated. Constructs were transfected into HeLa cells and cytotoxicity was measured 21 h later by LDH release assays. Cytotoxicity measurements are expressed relative to untagged, wild-type BteA. C. Western blot analysis of complex formation by full-length BteA^658^-FLAG (658 aa) and the non-cytoxic derivative BteA^644^-FLAG (644 aa) using α-FLAG monoclonal antibody. Both proteins were expressed in a Δ*bteA* derivative of *B. bronchiseptica* strain RB50. RB50 expressing untagged full-length BteA was used as a negative control. High-molecular-weight complexes (arrowheads) were observed for both BteA^658^-FLAG and BteA^644^-FLAG in whole-cell extracts and culture supernatants. Images shown are representative of three independent experiments.

A characteristic of the wild-type BteA protein is the induction of rapid, non-apoptotic cytotoxicity in a wide range of target cells (Stockbauer *et al*., 2003; Panina *et al*., 2005). In an attempt to identify a time interval where BteA might be detected and localized prior to the onset of cell death, we performed the time-course experiment shown in Fig. 2A.*bteA* was inserted downstream from the CMV promoter in plasmid pEGFP-N1, fusing EGFP to the C-terminus of BteA. Expression of the resulting fusion construct, BteA^658^-EGFP, was analysed by fluorescence microscopy at time intervals beginning 2 h after transfection. With the vector control, GFP-positive cells could be identified as early as 4 h., while no GFP-positive cells were found at any time point following transfection with the construct encoding BteA^658^-EGFP (Fig. 2B), and the fusion protein was not detectable by Western blot analysis (data not shown). Lactate dehydrogenase (LDH) release assays showed that expression of BteA with the C-terminal EGFP tag resulted in a level of cytotoxicity comparable to the native untagged protein (Fig. 2B). These data show that even trace amounts of BteA^658^-EGFP expression are sufficient to induce cytotoxicity and prevent detection of the fusion protein by fluorescence microscopy or Western blot analysis.

We reasoned that BteA derivatives with reduced cytotoxicity would be required for localization studies to proceed. A series of *bteA* fragments truncated at their 3′ termini were cloned into pEGFP-N1 to create C-terminal fusions with EGFP (Fig. 2B). Following transfection into HeLa cells, expression of BteA–EGFP derivatives was monitored by fluorescence microscopy and cytotoxicity was measured by LDH release assays (Fig. 2B). Although BteA^652^-EGFP retained full cytotoxicity, further truncations eliminated activity. The markedly reduced LDH release following transfection with BteA^644^-EGFP indicates that the last 14 aa of BteA are critical for full cytotoxicity. To determine if the BteA^644^ mutant retains structural features of the wild-type protein, we assessed its ability to self-associate, a characteristic of native BteA (Panina *et al*., 2005). Full-length BteA resolves as high-molecular-weight multimers on SDS-PAGE (Fig. 2C), a property that correlates with the formation of fibres in buffered aqueous solution that can be visualized by electron microscopy (data not shown). BteA^644^ retains this ability to form SDS-insoluble high-molecular-weight species, suggesting that it maintains its core structural scaffold despite a significant loss of cytotoxicity.

### BteA^644^ localizes to ezrin- and GM1-rich membrane sites

**Fig. 3:**
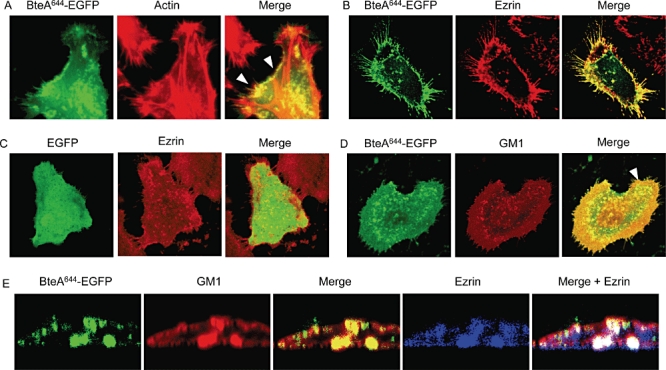
BteA localization. BteA^644^-EGFP was transfected into HeLa cells. Eighteen hours later cells were fixed, permeabilized and visualized by laser scanning confocal microscopy. Images represent average composite Z-stacks processed by Leica's LCS software. BteA^644^-EGFP colocalization was assessed using Alexa Fluor 568-conjugated phalloidin to stain actin (A), Alexa Fluor 633 α-ezrin (B and C) and Alexa Fluor 555-labelled CtxB to stain GM1 ganglioside (D). E. XZ scans of the region indicated by the arrow in (D). Colocalization appears as yellow for two-colour overlays, or white in three-colour overlay (4D), and is representative of a single Y section.

We next examined the localization of BteA^644^-EGFP in HeLa and L2 cells by scanning confocal microscopy. As shown in Fig. 3A, BteA^644^-EGFP clearly colocalized with cortical actin. BteA was observed to associate with regions of the cell surface (Fig. 3A, arrowheads), but not actin stress fibres, suggesting that it targets proteins and/or lipids positioned in the vicinity of actin-associated membrane sites. Cortical actin participates in the formation of signalling platforms with membrane receptor cytoplasmic domains (Xavier *et al*., 2004; Gajate and Mollinedo, 2005; Halayko and Stelmack, 2005; Chichili and Rodgers, 2009). These signalling complexes are often located at lipid raft microdomains anchored to the actin cytoskeleton by members of the ERM (ezrin-radixin-moesin) family of adaptor proteins. (Bretscher *et al*., 2002; Hoeflich and Ikura, 2004). We were thus interested to determine whether BteA colocalizes with ezrin, an ERM protein prominently expressed by HeLa cells. Cells transfected with constructs expressing BteA^644^-EGFP or EGFP alone were stained using antibodies against ezrin. Confocal microscopy revealed a high degree of colocalization between BteA^644^-EGFP and ezrin, but not EGFP and ezrin, suggesting that BteA is specifically targeted to ezrin-enriched membrane sites (Fig. 3B and C).

In many cells examined, BteA^644^-EGFP was particularly prominent in filopodia. Filopodia are rod-like projections on the cell surface that are supported by cortical actin and function in sampling and exploration of the environment (Fig. 3B) (Bryant, 1999; Small *et al*., 2002; Faix *et al*., 2009). BteA^644^ expression appeared to increase both the number and length of these structures in HeLa cells (Fig. 3B). In contrast, EGFP alone (Fig. 3C) did not localize to filopodia and did not result in abnormal filopodia formation. Due to their sensory and exploratory function, filopodia possess a large number of transmembrane receptors and raft-associated signalling complexes, which vary according to cell type and growth conditions (Lidke *et al*., 2005; Ladwein and Rottner, 2008). As filopodia usually contain high concentrations of lipid rafts, we next examined the colocalization of BteA with GM1 ganglioside, which serves as a marker for lipid rafts. Cells expressing BteA^644^-EGFP were stained using fluorescently labelled cholera toxin subunit B (CtxB), which binds with high avidity and specificity to ganglioside GM1 (Schon and Freire, 1989). Nearly complete colocalization was observed between BteA^644^-EGFP and GM1 (Fig. 3D). High-magnification XZ scans of the region indicated by the arrowhead in Fig. 3D further demonstrate that transfected BteA localizes precisely with GM1 in cell membranes (Fig. 3E).

### BteA contains a conserved, multifunctional N-terminal domain

**Fig. 4:**
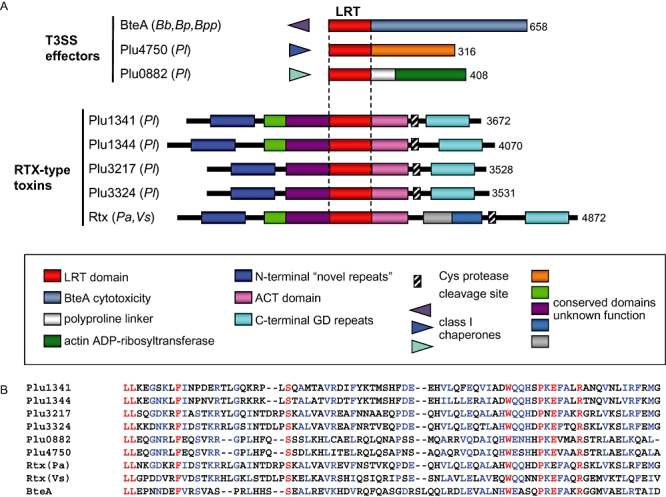
Distribution of LRT domains in putative bacterial virulence factors. A. Schematic representation of known and predicted proteins with LRT domains (red bars) and their closest homologues lacking LRT sequences. The schematic is not drawn to scale. Homologous domains are coloured as indicated. The cysteine protease domain in RtxA and related toxins, and a cleavage site identified in RtxA, are indicated by hatched boxes and an arrow respectively (Sheahan *et al*., 2007) (see *Discussion*). Species origin is indicated in parentheses by a two-letter code: Bb, *B. bronchiseptica*; Bp, *B. pertussis*; Bpp, *B. parapertussis*; Pl, *P. luminescens*; Pa, *Photorhabdus asymbiotica*, Vs, *V. splendidus*. B. Alignment of LRT domains in known and predicted virulence factors. The central region of the BteA LRT domain (aa 34–112) is shown, with identical amino acids present in all nine sequences in red and conserved residues in blue.

The BteA proteins expressed by *Bordetella* species are highly homologous, especially within an N-terminal 130 aa segment (Fig. 1A). Using iterative psi-blast, six homologues of this domain were found in *Photorhabdus luminescens* and two others in *Vibrio splendidus* and *Photorhabdus asymbiotica* (Fig. 4A and B). We had previously predicted that two of the *P. luminescens* proteins (Plu0822 and Plu4750) function as T3SS effectors on the basis of their close proximity to class I chaperones (Panina *et al*., 2005). The remaining four *P. luminescens* proteins (Plu1341, Plu1344, Plu3217, Plu3324) and the proteins from *V. splendidus* and *P. asymbiotica* are homologous to RtxA, an atypical RTX toxin with actin cross-linking activity (Lin *et al*., 1999; Fullner and Mekalanos, 2000).

**Fig. 5:**
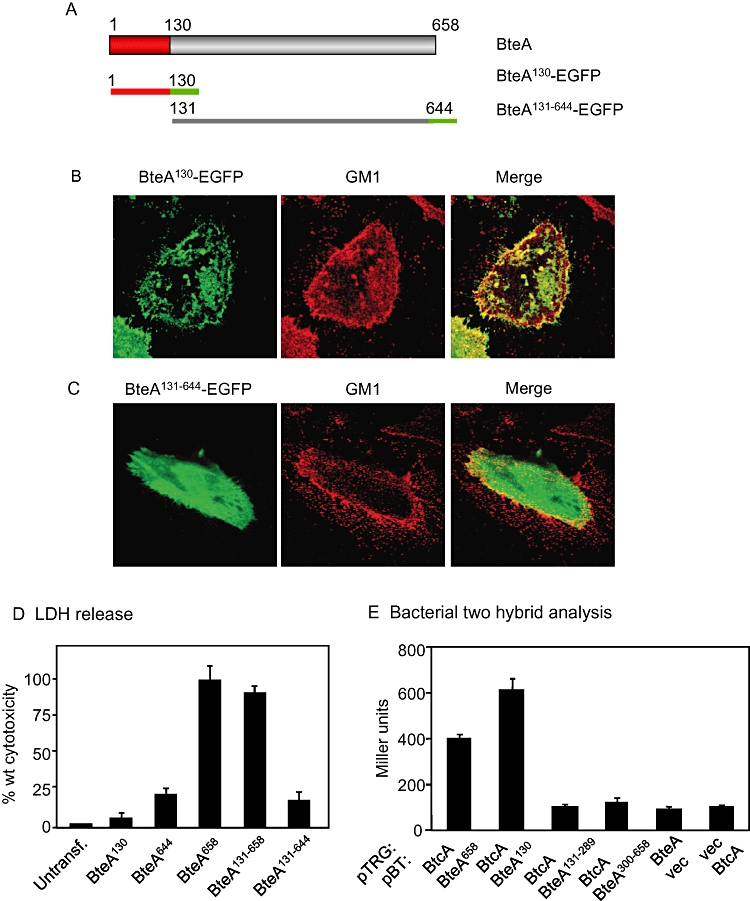
The N-terminal LRT domain of BteA mediates chaperone binding and host cell localization. A. Constructs used in this experiment. Notations are the same as in Fig. 3A. HeLa cells were transfected with BteA^130^-EGFP (B) or BteA^130-644^-EGFP (C). EGFP fusion proteins and Alexa Fluor 555-labelled CtxB bound to GM1 were visualized by confocal microscopy. Images are single Z sections. Colocalization between transfected proteins and GM1 appears as yellow on the overlay images. D. Cytotoxicity assays. Full-length (BteA^658^) or truncated BteA–EGFP fusion constructs were transfected into HeLa cells and LDH release was measured 21 h post transfection. Cytotoxicity is expressed as a percentage relative to wild-type BteA. E. Bacterial two-hybrid analysis of interactions between BtcA, BteA and BteA domains. The pTRG target vector alone (vec), or pTRG expressing full-length BtcA fused to the amino-terminal domain of the alpha subunit of RNA polymerase was transformed with the pBT bait vector (vec), or pBT expressing full-length BteA (BteA^658^), or fragments truncated at their N- and/or C-termini fused to the C-terminus of the lambda repressor.

Given the presence of homologous sequences in known and putative virulence determinants from multiple species, we hypothesized that the N-terminal 130 aa segment of BteA may perform a conserved cellular function, possibly by directing localization. This was tested by fusing the first 130 aa of BteA to EGFP (BteA^130^-EGFP) and examining transfected cells by confocal microscopy. BteA^130^-EGFP localized to GM1-positive lipid rafts in a manner indistinguishable from BteA^644^-EGFP (Figs 3D and 5B respectively). Similarly, the localization patterns of all C-terminal truncated derivatives of BteA (Fig. 3B) were indistinguishable from BteA^644^-EGFP, while the first 30 aa of BteA was unable to direct EGFP to membrane sites (data not shown). When a construct lacking the N-terminal 130 aa region of BteA (BteA^131-644^-EGFP) was tested, it too failed to localize to GM1-stained raft microdomains (Fig. 5C). These observations demonstrate that an LRT signal of BteA is contained between aa 1 and 130. Although the BteA LRT motif is essential for localization, it is not required for cytotoxicity following transfection (Fig. 5D). Expression of BteA^131-658^-EGFP conferred a high level of cytotoxicity, even though there was no detectable localization to the cell membrane. Although overexpression of toxic domains may compensate for the lack of localization following transfection, membrane association is likely to be important during T3SS-mediated translocation (see *Discussion*).

N-terminal sequences of T3SS effectors contain secretion signals and often mediate chaperone–effector interactions (Harrington *et al*., 2003). *bteA* is directly adjacent to a divergently transcribed locus, *btcA*, which is predicted to encode a class I chaperone (Panina *et al*., 2005). As shown in Fig. 5E, full-length BteA associates with BtcA in a bacterial two-hybrid system (Dove and Hochschild, 2004) and the 130 aa LRT domain is both necessary and sufficient for this interaction. Taken together, our results show that the N-terminus of BteA is multifunctional, mediating chaperone interactions and directing the localization of BteA to lipid raft microdomains in eukaryotic cells.

### BteA fractionates with detergent-resistant membranes

**Fig. 6:**
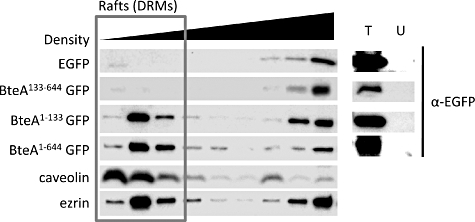
BteA is present in DRM fractions from transfected cells. HeLa cells were transfected with the constructs indicated (left sidebar), and fractionated as described in *Experimental procedures*. Western blots were probed with α-EGFP or antibodies to ezrin and caveolin as indicated in the left sidebar. Transfected (T) or untransfected (U) cell controls are shown in the right sidebar. Lanes within the red outline are low-density DRM fractions.

The relative insolubility of cholesterol in Triton X-100 facilitates the separation of cholesterol-enriched lipid rafts during cell fractionation (Ignoul *et al*., 2007). Detergent-resistant membrane (DRM) fractions were prepared from HeLa cells transfected with BteA–EGFP derivatives to further investigate the association between BteA and lipid rafts. HeLa cells expressing EGFP, BteA^644^-EGFP, BteA^130^-EGFP or BteA^130-644^-EGFP were fractionated by Triton X-100 solubilization followed by sucrose gradient density centrifugation. Cellular fractions were separated by SDS-PAGE and Western blots were probed with antibody to EGFP. As expected, fusion constructs containing the BteA LRT domain (BteA^644^- and BteA^130^-EGFP) were abundant in the low-density Triton X-100-insoluble DRM fractions, suggesting that BteA associates with rafts, raft proteins or associated protein complexes (Fig. 6). Notably, the distribution pattern of BteA in DRMs was nearly identical to that of ezrin and the lipid raft marker, caveolin-1. Although clearly associated with lipid rafts, the presence of detectable levels of LRT-containing BteA derivatives, ezrin and caveolin in dense cellular fractions is likely due to the presence of non-membrane-associated intracellular pools and/or disruption of complexes during fractionation. In contrast, BteA^130-644^-EGFP and the EGFP-negative control were exclusively present in dense, non-membrane-associated Triton X-100-soluble fractions. Thus, the LRT domain-dependent fractionation of BteA within DRMs coincides with results from colocalization studies.

### BteA is translocated to the cytoplasmic face of membrane raft locations that function as sites for bacterial attachment

**Fig. 7:**
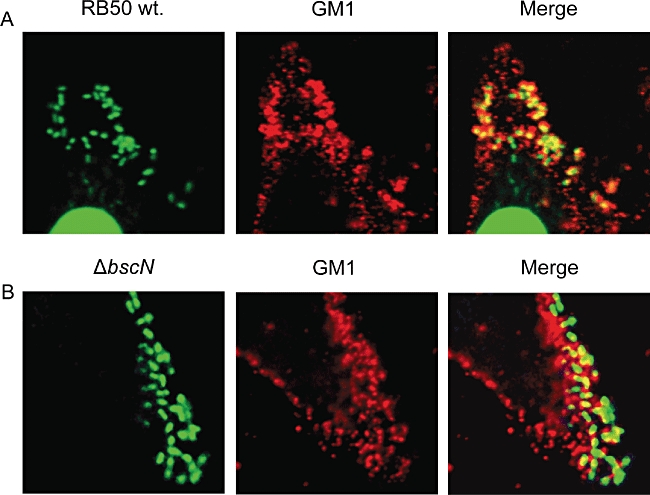
*B. bronchiseptica* RB50 adheres to GM1-containing lipid rafts. Images are single Z sections, and are representative of at least three separate experiments. HeLa cell monolayers were infected with *B. bronchiseptica* strain RB50 (A) or the T3SS-defective strain RB50 Δ*bscN* (B) for 25 min, washed, fixed and stained. DAPI staining (green) reveals bacteria adherent to HeLa cells. GM1 appears red, and overlap is yellow in the merged images.

The observation that BteA targets discrete locations on host cell membranes prompted us to examine whether a correlation exists with sites of *Bordetella* attachment. HeLa cells were infected with wild-type *B. bronchiseptica* strain RB50 for 15 min, a time point before cytotoxicity is observed. Cells were washed to remove unbound bacteria, fixed and stained with CtxB. Remarkably, almost every cell-associated bacterium was found to colocalize with GM1-positive rafts (Fig. 7A). We reasoned that *B. bronchiseptica* may specifically adhere to lipid rafts and that BteA functions locally following injection, or that BteA and/or other T3SS-associated factors actively recruit GM1-rich structures to sites of *Bordetella* attachment. To distinguish these possibilities we analysed the attachment pattern of a T3SS ATPase mutant, RB50 Δ*bscN* (Yuk *et al*., 1998), which adhered to GM1-enriched sites in a manner indistinguishable from wild-type RB50 (Fig. 7B). Association with lipid rafts therefore is independent of T3SS activity and is likely mediated by adhesin specificity.

**Fig. 8:**
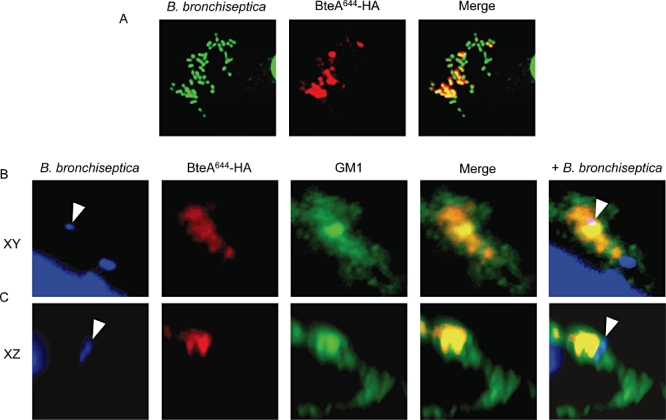
BteA is translocated by the *bsc* T3SS into lipid rafts that coincide with sites of bacterial attachment. A single HeLa cell is shown, infected with RB50 expressing an HA-tagged BteA^644^ (BteA^644^-HA) and stained using DAPI (bacteria) and antibody to HA. A. BteA^644^-HA (red) colocalizes with adherent bacteria (green). B. 100× magnification, XY confocal image of BteA^644^-HA stained with DAPI for bacteria (blue), α-HA (red) and GM1 ganglioside (green). C. 100× magnification XZ scan and merged images of the same portion of the cell as in (B). The arrows indicate the position of bacterial cells. Colocalization appears yellow in two colour overlays, and white in triple colour overlays.

We next determined if BteA localizes to lipid rafts following T3SS-mediated translocation by adherent *Bordetella*. HeLa cells were infected with RB50 derivatives expressing haemagglutinin (HA)-tagged BteA (BteA^644^-HA), permeabilized with Triton X-100 and stained with DAPI, CtxB and an α-HA monoclonal antibody. BteA^644^-HA closely associated with adherent bacteria (Fig. 8A) and colocalized with GM1-positive lipid rafts (Fig. 8B). XZ confocal scans of the region marked in Fig. 8B (arrow) show that BteA is translocated into lipid rafts that coincide with sites of bacterial attachment (arrowheads, Fig. 8C). T3SS-translocated BteA^644^-HA could not be visualized when the Triton X-100 membrane permeabilization step was omitted, indicating that BteA was not localized to the outer surface of the plasma membrane (data not shown). Deletions in loci encoding the BscN ATPase (RB50 Δ*bscN*), or the BopB translocon component (RB50 Δ*bopB*), eliminated the ability to detect raft-associated BteA^644^-HA, demonstrating a requirement for T3SS-mediated translocation (data not shown). These data demonstrate that translocated BteA associates with the cytoplasmic face of lipid rafts that serve as attachment sites for *Bordetella*, and suggest that BteA acts locally following bacterial attachment. It is interesting to note that only a subset of adherent bacteria are associated with translocated BteA at any given time (Fig. 8A). The mechanistic basis for this lack of synchrony is currently unknown.

### Disruption of lipid rafts decreases T3SS-induced cytotoxicity

**Fig. 9:**
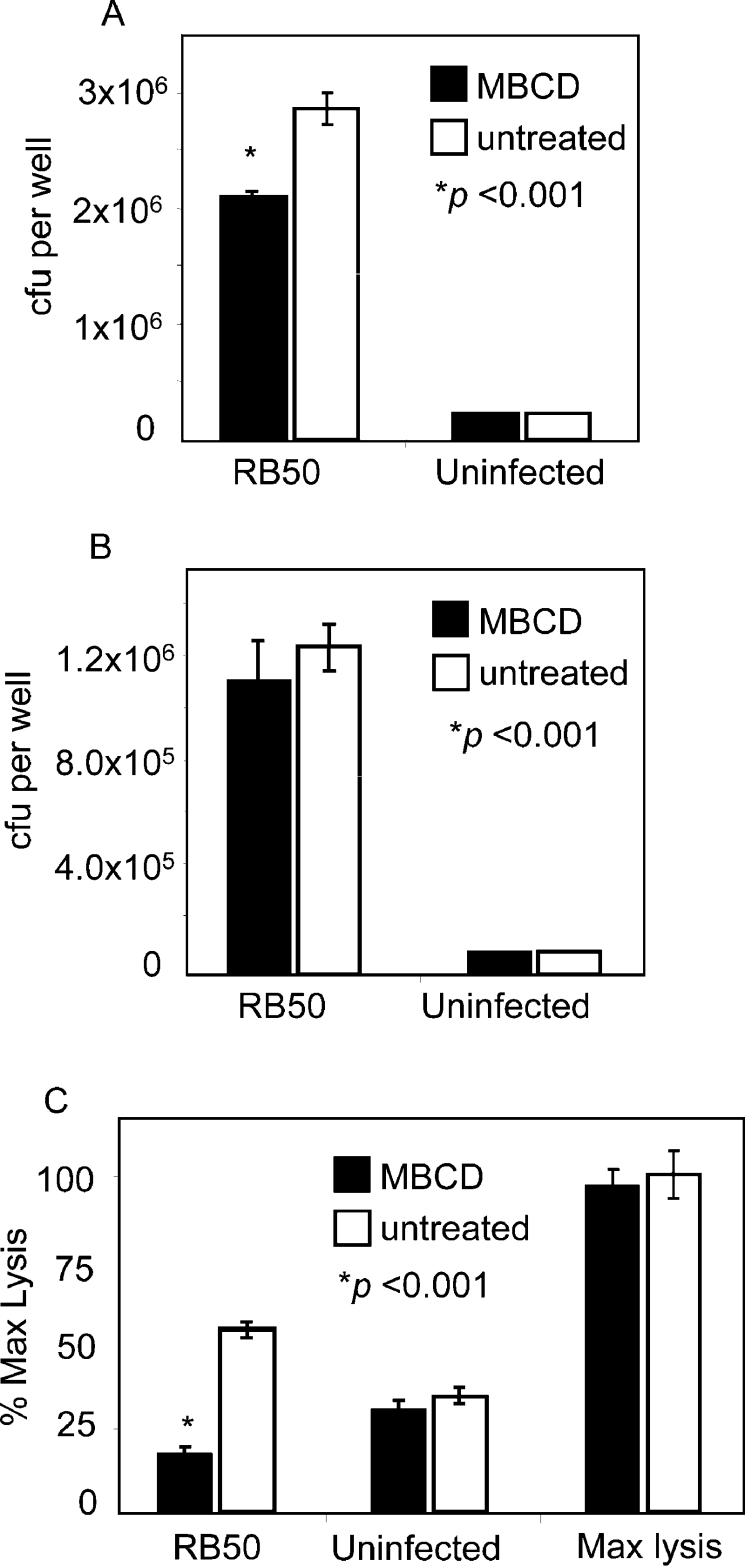
MBCD treatment interferes with bacterial attachment and protects cells from T3SS-mediated cytotoxicity. A. HeLa cells incubated with media alone (black bars) or 2.5 mM MBCD (grey bars) for 30 min were washed and infected with wild-type *B. bronchiseptica* RB50 for 15 min at a multiplicity of infection of 50:1. Following infection, cells were extensively washed, solubilized and adherent bacteria measured as colony forming units (cfu) per well. All statistical analyses were performed using the Student's *t*-test with data from three independent experiments. Asterisk indicates a *P*-value < 0.001. B. HeLa cells were first infected with wild-type *B. bronchiseptica* RB50 for 15 min, washed and incubated with media alone (black bars) or 2.5 mM MBCD (grey bars) for 30 min Cells were extensively washed, solubilized, and adherent bacteria measured as cfu per well. There was no statistically significant difference between untreated and MCDB-treated cells. C. HeLa cells infected with *B. bronchiseptica* RB50 for 15 min, or uninfected, were incubated with media alone (black bars) or 2.5 mM MBCD (striped bars) for 30 min as described in (B). Cells were then washed and incubated in fresh media for 2 h. Bars indicate cytotoxicity measured by LDH release as a percentage of maximum cell lysis. Wells designated as ‘Max lysis’ were treated with MBCD as described above and lysed with 0.9% Triton-X100 to generate maximum values for LDH release.

As both *B. bronchiseptica* and BteA preferentially localize to lipid rafts, we were curious to determine if the integrity of cholesterol-enriched membrane domains is important for attachment and/or T3SS-mediated cytotoxicity. Methyl-β-cyclodextrin (MBCD), a cyclic glucose oligomer that forms soluble inclusion complexes with cholesterol, was used to disrupt lipid rafts (Shaw, 2006). HeLa cells were pretreated with 2.5 mM MBCD or media alone for 30 min, washed and briefly infected with *B. bronchiseptica* RB50. Mild treatment conditions were employed to minimize the MBCD-dependent cytotoxicity observed with higher concentrations or longer exposure. As shown in Fig. 9A, a modest decrease in bacterial attachment was observed at 20 min, a time before T3SS-induced cytotoxicity could be detected. In the experiment in Fig. 9B, HeLa cells were first infected with *B. bronchiseptica* RB50 for 15 min, followed by a 30 min treatment with MBCD or media alone. In this case, no significant difference was observed between MBCD-treated cells and untreated controls, indicating that treatment with MBCD has little effect on previously attached *Bordetella.*

Having identified a MBCD treatment protocol that was non-toxic and did not disrupt bacterial attachment, we next determined the effects of cholesterol depletion on T3SS-induced cytotoxicity. HeLa cells were infected with *B. bronchiseptica* RB50 for 15 min, treated with MBCD or media alone for 30 min, washed extensively and the infection was allowed to proceed for a total of 2 h. As shown in Fig. 9C, cytotoxicity was reduced to background levels in cells subjected to mild treatment with MBCD. These results suggest that BteA-mediated cytotoxicity following *Bordetella* infection is dependent on the integrity of lipid rafts.

### Functional conservation of LRT domains in T3SS effectors and RTX toxins

**Fig. 10:**
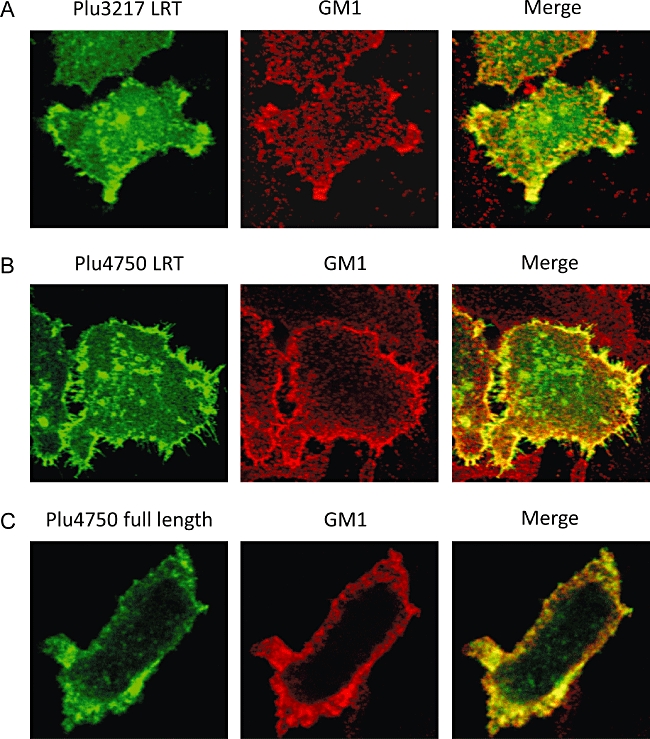
Functional conservation of LRT domains from T3SS effectors and RTX-type toxins. Colocalization of ezrin with the isolated LRT domains from Plu3217 (A) and Plu4750 (B) fused to EGFP, and with the full-length Plu4750 protein fused to EGFP (C). HeLa cells were transfected with expression plasmids for EGFP fusion proteins, stained for GM1 and visualized by confocal microscopy. Colocalization between transfected proteins and GM1 appears as yellow on the merged images. Images are single Z sections.

The relative positions of LRT domains differ according to the known or predicted nature of the protein; T3SS effectors bear them near their N-termini while RTX-type toxins encode them near the middle of their 3500- to 4900-aa-long sequences (Fig. 4A). Having demonstrated that the BteA LRT domain is necessary and sufficient for membrane localization, we determined whether putative LRT sequences from two *P. luminescens* ORFs, one predicted to encode a T3SS effector (Plu4750) and the other an RTX toxin (Plu3217), were capable of directing EGFP fusion constructs to lipid rafts. Sequences encoding the *P. luminescens* LRT domains were fused to EGFP at their C-termini and transfected into HeLa cells. As predicted, both LRT sequences localized the EGFP fusion constructs to ezrin-rich sites, despite their presence in otherwise unrelated proteins (Fig. 10A–C). Moreover, the same localization pattern was observed when full-length Plu4750 was fused to EGFP, confirming that the LRT motif functions as predicted in the context of the entire protein (Fig. 10C). On the basis of these observations we conclude that the N-terminal LRT motif of BteA defines a newly identified family of localization domains that target diverse bacterial proteins to membrane rafts in eukaryotic cells.

## Discussion

BteA is the only *Bordetella* T3SS effector protein described to date (Panina *et al*., 2005). This stands in marked contrast to other T3SS-expressing species where numerous effectors have been identified by proteomic, bioinformatic or translocation screens (Fouts *et al*., 2002; Chang *et al*., 2005). BteA is both necessary and sufficient for the induction of rapid cell death in a remarkably broad range of cell types (Panina *et al*., 2005). Although BteA has no full-length homologues among previously characterized proteins, it is highly conserved and functionally interchangeable between *Bordetella* species. Despite its central role in virulence, the molecular function and intracellular targets of BteA have remained elusive.

The potent cytotoxicity of BteA precludes the ability to detect even trace amounts of full-length protein in mammalian cells. A deletion of 14 aa from the C-terminus was sufficient to eliminate cytotoxicity. This derivative, BteA^644^, retained the ability to form high-molecular-weight, detergent-insoluble complexes that were similar to full-length BteA, suggesting that the structural scaffold of the protein remains intact. Most importantly, the reduction of cytotoxicity permitted the efficient detection of intracellular protein. Following transfection or T3SS-mediated translocation, BteA^644^ clearly localized to ezrin- and GM1-rich membrane microdomains in HeLa cells as observed by confocal microscopy. Localization was dependent on the 130 aa LRT motif present at the N-terminus of BteA. Identical patterns of localization were observed with BteA derivatives that retain the LRT sequence, and its removal disrupted normal targeting. As predicted, BteA associated with low-density, detergent-insoluble membrane fractions from transfected cells, indicating direct or indirect association with lipid rafts. Importantly, BteA displayed a pattern of localization following T3SS-mediated translocation that was identical to that observed following transfection. When sites of *Bordetella* attachment were examined, nearly every cell-associated bacterium was found to colocalize with GM1 and there was a striking correlation between the position of extracellular bacteria and the location of translocated BteA. These observations tempt the hypothesis that BteA modulates localized cellular responses to *Bordetella* infection, and we speculate that this may be especially relevant to interactions with phagocytic antigen-presenting cells. This is consistent with previous results showing T3SS-dependent modulation of T cell- and B cell-mediated responses during *B. bronchiseptica* infections (Skinner *et al*., 2005; Pilione and Harvill, 2006; Siciliano *et al*., 2006).

Although lipid raft localization is likely to be essential under physiologically relevant conditions, a BteA^130-658^ construct lacking LRT sequences displayed cytotoxic activity following transfection. This contrasts with observations that transfected and translocated BteA behave in an otherwise identical manner and that depletion of membrane cholesterol with MBCD abrogates BteA-dependent cell death following T3SS-mediated delivery. A likely explanation is that the LRT domain plays an essential role in facilitating interactions between BteA and host cell targets during T3SS-mediated translocation, and overexpression of BteA following transfection eliminates the need for LRT-dependent localization. In addition to its role in membrane association, the BteA LRT domain also contains sequences that mediate secretion and chaperone binding. The identification of mutations that disrupt targeting without affecting secretion or translocation will facilitate further studies on the importance of LRT for BteA function. Experiments aimed at identifying BteA binding partners responsible for localization and/or cytotoxicity are also underway. On the basis of results presented here, we expect that BteA targets will be associated with the host cell membrane and cholesterol-rich domains. Likely candidates include components of membrane-associated signalling complexes that are found in lipid rafts and are known to be associated with the cortical actin cytoskeleton.

The BteA LRT domain represents a newly identified motif capable of directing proteins to lipid rafts. Although the identification of similar sequences at the N-termini of other T3SS effectors was not unexpected, we were intrigued to find homologous motifs in RTX-type toxins. A model has been recently proposed for *Vibrio cholerae* RtxA, which postulates that both N-terminal and C-terminal sequences associate with the plasma membrane and mediate translocation of the central portion of the protein into the cytosol. A cysteine protease domain then cleaves the toxin between L3428 and A3429 (Fig. 4A) and additional cleavage events liberate the actin cross-linking and Rho-inactivation domains from their membrane anchors (Sheahan *et al*., 2007; Prochazkova and Satchell, 2008). As homologous cysteine protease domains are found in all of the RTX-type toxins shown in Fig. 4A, proteolytic processing following cytosolic delivery is likely to be a common feature (Sheahan *et al*., 2007). For the *P. luminescens* and *V. splendidus* RTX-type toxins, we propose that adjacent LRT motifs target activity domains released by proteolysis to GM1-rich lipid rafts. The relative positions of cleavage sites would determine exactly which domains colocalize with the LRT. Although LRT-containing proteins represent distinct classes of virulence determinants with completely different translocation mechanisms, the ability of the LRT domains from a *Photorhabdus* effector and an RTX-type toxin to guide fusion proteins to lipid rafts clearly demonstrates conserved function.

*Photorhabdus luminescens* is a bacterial symbiont of entomopathogenic nematodes. It is transported to the hemocoel of an insect prey, which is then killed by an arsenal of toxins and T3SS effectors (Derzelle *et al*., 2004). *V. splendidus* is a dominant *Vibrio* in coastal sediments and seawater, which is pathogenic for bivalves and fish and shares an ecological niche with *V. cholerae* (Lacoste *et al*. 2001, Gay *et al*., 2004). *B. bronchiseptica* infects the respiratory epithelia of a wide range of mammals, including humans, and appears to have an environmental reservoir as well (Akerley *et al*., 1995). The evolutionary journey the LRT domain has taken between pathogens of insects, fish and mammals provides a poignant example of the conservation of virulence mechanisms in nature and exemplifies the ancient origins of bacterial–host interactions.

## Experimental procedures

### Bacterial strains and cell culture

HeLa cells were maintained in DMEM (Gibco) supplemented with 10% Fetal Bovine Serum (FBS, Hyclone) and penicillin-streptomycin. L2 cells were maintained in DMEM or F12K media (Invitrogen) supplemented with 10% FBS and pen-strep. Both cell lines were grown at 37°C in a 5% CO_2_ atmosphere. *B. bronchiseptica* strains RB50, RB50 Δ*bscN*, Δ*bteA and*Δ*bopB* have been previously described (Cotter and Miller, 1994; Yuk *et al*., 1998; Panina *et al*., 2005). Full-length or truncated *bteA* loci from *B. bronchiseptica*, *B. pertussis* Tohama I or *B. parapertussis* strain 12822 (Parkhill *et al*., 2003) were cloned into pBBR1MCS-5 for expression in *Bordetella*. All *B. bronchiseptica*-derived strains were cultured in Stainer-Scholte liquid medium or on Bordet-Gengou agar medium (Becton Dickinson Microbiology Systems, San Jose, CA) containing 7.5% defibrinated sheep blood (Mission Laboratories, Rosemead, CA) and appropriate antibiotics for plasmid selection.

### Construction of BteA–EGFP and LRT-EGFP fusions

Genes encoding full-length BteA (BteA^658^) and BteA derivatives with C-terminal deletions (BteA^30^, BteA^300^, BteA^400^, BteA^500^, BteA^540^, BteA^580^, BteA^620^, BteA^628^, BteA^644^ and BteA^652^) were cloned into the NheI and HindIII restriction sites in pEGFP-N1 (Clontech), in-frame with the C-terminal EGFP tag. *bteA* fragments were PCR-amplified from *B. bronchiseptica* RB50 using Herculase (Stratagene). All fragments include the first codon of BteA and terminate at the codon indicated by their superscript designation (e.g. BteA^300^ includes codons 1–300). EGFP-tagged mutants containing N-terminal BteA deletions (BteA^131-644^, BteA^131-658^) were constructed by cloning BteA fragments into the same sites on pEGFP-N1 and adding ATG start codons for translation initiation. LRT domains from Plu4750 (codons 1–132), Plu3217 (codons 1808–1907) and the full-length Plu4750 protein (codons 1–316) were cloned from *P. luminescens* sp*. luminescens* (ATCC) into pEGFP-N1 to construct EGFP fusions. An N-terminal ATG codon was added immediately upstream of LRT sequences from Plu3217.

### Cytotoxicity assays, transfection and MBCD treatments

*Bordetella bronchiseptica* strains expressing or *bteA*_*Bb*_*, bteA*_*Bp*_ or *bteA*_*Bp*p_ alleles grown to mid-log phase were added to cell cultures at a multiplicity of infection of 50:1 and centrifuged onto cultured cells at 640 *g* for 5 min. DMEM + 1% FBS was added to cells following a thorough wash with PBS, and incubated at 37°C for indicated periods of time. Cytotoxicity was assayed with the CytoTox 96 LDH release assay kit (Promega) (Lappalainen *et al*., 1994) following the manufacturer's suggested procedures. To analyse cytotoxicity from transfected constructs, HeLa or L2 cells grown in 12-well plates were transfected with 1.6 μg of plasmid DNA using Lipofectamine LTX (Invitrogen). Transfected cell cultures were briefly centrifuged to remove cell debris, and 50 μl of media was removed and assayed for LDH release at various times post transfection. For MBCD protection or attachment assays, HeLa cells were washed with PBS and incubated in DMEM + 2.5 mM MBCD (Sigma Aldrich) for 30 min. MBCD-containing medium was removed, cells were washed with PBS and cultured in reduced serum DMEM (1.0% FBS) lacking MBCD. Fifteen minutes after centrifugation, colony-forming units of attached bacteria were assayed by spreading serial dilutions of the cell lysate on BG agar medium following washing and solubilization of the cell monolayer in 0.2% Triton X-100. *Bordetella*-induced cytotoxicity was measured as described above at 2 h post infection.

### Confocal microscopy

Cells were grown in multiwell plates on glass coverslips and transfected as described above. Eighteen hours post transfection, cells were washed and fixed with 4% paraformaldehyde in 1× PBS for 30 min at room temperature. To visualize EGFP fusion proteins, glass slides were mounted using Vectashield mounting media (Vector Laboratories) and sealed with clear nail polish. For actin and ezrin staining, cells were permeabilized with 0.1% Triton X-100 for 10 min, and stained with either Alexa Fluor-conjugated phalloidin (Invitrogen) or α-ezrin primary antibody (BD Biosciences) followed by fluorescently labelled secondary antibody (Invitrogen). GM1 staining using Alexa Fluor 555-labelled CtxB was performed as described (Chen *et al*., 2007). HeLa cells infected with *B. bronchiseptica* were washed twice with PBS, fixed as described above, and *B. bronchiseptica* was visualized using Vectashield mounting media with DAPI (Vector Laboratories). All images were acquired with a Leica SP2-AOBS laser scanning confocal microscope set-up. Images shown in the figures are representative of at least three independent experiments and were produced with Leica's LCS confocal software.

### Western blot analysis

*Bordetella bronchiseptica* strains were grown overnight in Stainer-Scholte medium and cell cultures were separated into pellet and supernatant fractions. Whole-cell extracts were prepared by dissolving pellets in Laemmli buffer (Laemmli, 1970). Proteins from supernatant fractions were precipitated with 10% trichloroacetic acid for 6 h at 4°C. OD equivalents of *B. bronchiseptica* whole-cell lysates and supernatants were separated on 10% SDS-PAGE gels and transferred to PVDF membranes (Millipore). Membranes were incubated with mouse α-FLAG antibody (Sigma Aldrich) and antigen–antibody complexes were detected with horseradish peroxidase-conjugated α-mouse immunoglobulin (Amersham Biosciences) and visualized by chemiluminescence using SuperSignal West Pico chemiluminescent substrate (Pierce, Rockford, IL).

### Bacterial two-hybrid analysis

*Bordetella bronchiseptica* sequences were cloned into the BacterioMatch (Stratagene) two-hybrid ‘bait’ vector pBT, creating fusions with the C terminus of the lambda cI protein, and/or into the cognate ‘target’ vector pTRG, creating fusions with the amino-terminal domain of the alpha subunit of RNA polymerase. Clones were confirmed by sequence analysis and cotransformed into the BacterioMatch I reporter strain (XL1Blue MRF′*laqI*^q^*bla lacZ* Kan^r^). To assay β-galactosidase activity, overnight cultures were subcultured to an optical density (OD_600_) of 0.07 in LB medium containing tetracycline (15 μg ml^−1^) and chloramphenicol (34 μg ml^−1^). Cultures were incubated at 30°C until the OD_600_ reached 0.4, at which point isopropyl β-D-thiogalactoside was added to a final concentration of 10 μM. After 2 h of isopropyl β-D-thiogalactoside induction, cells were harvested and permeabilized with SDS and CHCl_3_, and β-galactosidase activity was measured using the method described by Miller (Sommer *et al*., 1978).

### DRM fractionation

The DRM fractions were prepared from HeLa cells as described (Ignoul *et al*., 2007). Approximately 18 h after transfection with BteA- and LRT-fusion derivates, 1–2 × 10^7^ cells were washed with PBS, scraped into 1 ml of lysis buffer containing 1.0% Triton X-100 and protease inhibitor cocktail (Roche), and adjusted to a final sucrose concentration of 45%. The cell lysate was overlaid with a 30–5% sucrose step gradient, and separated for 16 h at 38 000 r.p.m. by isopycnic floatation in a Beckman SW-41 rotor. 1 ml fractions were collected and sedimented in a Ti70.1 rotor at 40 000 r.p.m. for 1 h. The pellets were resuspended in 1× Laemmli buffer (Laemmli, 1970) and electrophoresed on 4–16% gradient SDS-PAGE gels (Bio-Rad). Proteins were transferred to PVDF-Plus membranes (Amersham) and probed with rabbit α-EGFP (Abcam), mouse α-ezrin (BD-Biosciences) or mouse α-caveolin-1 (Santa Cruz). Chemiluminescent detection was performed using Amersham's ECL reagents and Kodak BioMax MS film.

### Bacterial attachment assays

HeLa cells pre-incubated with MBCD or media alone as described above were infected with *B. bronchiseptica* RB50 at a multiplicity of infection of 50:1 for 25 min. Infected cultures were briefly centrifuged at 640 *g* for 5 min followed by a thorough wash with PBS. Cells were solubilized in 0.2% Triton X-100, and aliquots assayed for colony-forming units on BG agar medium. In parallel, attached *B. bronchiseptica* were stained, visualized by light microscopy, and manually counted.
